# Case report: Good response to CMOP regimen containing mitoxantrone hydrochloride liposome (PLM60) as induction chemotherapy in patients with angioimmunoblastic T-cell lymphoma

**DOI:** 10.3389/fonc.2024.1331154

**Published:** 2024-01-31

**Authors:** Lijie Liang, Ming Jiang

**Affiliations:** Department of Medical Oncology, Cancer Center, West China Hospital, Sichuan University, Chengdu, China

**Keywords:** angioimmunoblastic T-cell lymphoma, mitoxantrone hydrochloride liposome, PLM60, CMOP, case report

## Abstract

Angioimmunoblastic T-cell lymphoma (AITL) is a highly aggressive subtype of peripheral T-cell lymphoma. The current prognosis with the first-line standard of care remains unsatisfactory, necessitating the exploration of more effective treatment options. We reported 5 cases of AITL receiving CMOP (mitoxantrone hydrochloride liposome, cyclophosphamide, vincristine, and prednisone). Cases 1 and 2 initially received CHOP as first-line induction therapy but switched to CMOP due to inadequate efficacy and cardiac adverse events. Cases 3, 4, and 5 were newly diagnosed and received CMOP. All patients achieved complete remission with acceptable cardiotoxicities and hematologic toxicities. After study treatment discontinuation, Cases 1 and 3 underwent autologous stem cell transplantation, and Cases 4 and 5 received oral maintenance agents. At the last follow-up, 4 patients remained in remission and 1 (Case 2) exhibited tumor recurrence. CMOP showed promise as a potential treatment option for AITL patients. Further research is essential to identify its efficacy and safety.

## 1 Introduction

Angioimmunoblastic T-cell lymphoma (AITL) is a rare and aggressive peripheral T-cell lymphoma (PTCL), accounting for 1%-2% of all non-Hodgkin lymphomas (1). Disease relapse occurred commonly in the majority of AITL patients after a short-term remission with a 5-year overall survival (OS) rate of 44% and a progression-free survival (PFS) rate of 32% (2). Additionally, AITL often shows severe systemic symptoms including fever, weight loss, and lymphadenopathy, seriously affecting the quality of life (3). At present, the standard of care for AITL patients remains unclear. CHOP-like (cyclophosphamide, doxorubicin, vincristine, prednisone), anthracycline-based ± etoposide, presented a conventional first-line treatment for AITL (4). However, newly diagnosed AITL showed a poor response to CHOP with a complete remission (CR) rate of 27.6% (5). Therefore, the development of novel therapeutic options is in urgent need for improving clinical benefits for patients with AITL.

Mitoxantrone, as a potential treatment option for various malignancies, was unable to be widely used due to cardiotoxicity (6, 7). Mitoxantrone hydrochloride liposome injection (PLM60) improved the pharmacokinetics parameters and tissue distribution characteristics in the human body by a liposomal encapsulation form (8), thus reducing the normal tissue damage and cardiotoxicity associated with anthracene drugs (9). In addition, PLM60 selectively accumulates in tumors and continuously releases drugs within the tumor microenvironment (10), providing enduring benefits. In a phase II trial, PLM60 monotherapy showed promising efficacy with a CR rate of 23.1% and an acceptable safety profile in relapsed/refractory AITL (11), highlighting the potential for further exploration in combination therapies. The combination of PLM60, cyclophosphamide, vincristine, and prednisone (defined as the CMOP regimen) in our study might provide more clinical benefits for AITL.

Here, we presented the clinical histories of 5 patients, the reason for selecting CMOP, treatment responses, and implications for AITL management. This report showed the potential benefits of the CMOP regimen containing PLM60 in AITL patients, with encouraging efficacy and an acceptable safety profile.

## 2 Case presentations

We reported 5 patients with AITL, treated at the West China Hospital of Sichuan University. The clinical features of the five cases were included in **Table 1**. All 5 patients exhibited durable responses and good tolerance to the 28-day cycle CMOP regimen. The last follow-up date was in August 2023.

**Table 1:** Case characteristics.

	Case 1	Case 2	Case 3	Case 4	Case 5
Age (year)/sex	50/F	66/F	53/M	54/M	52/F
ECOG PS	1	2	1	1	3
B symptoms	Positive	Negative	Negative	Positive	Negative
Ann Arbor Stage	IV	II	II	III	IV
Bulky disease >5 cm	Negative	Negative	Negative	Negative	Negative
Extranodal sites ≥2	Positive	Negative	Positive	Negative	Positive
Bone marrow involvement	Negative	Negative	Negative	Negative	Positive
LDH	Elevated	Normal	Normal	Normal	Elevated
β2-MG	Elevated	Elevated	Normal	Elevated	Elevated
Platelets<150000/mm^3^	Negative	Positive	Negative	Negative	Positive
EB DNA	7780	Negative	130	455	718
IPI	3	1	1	1	4
PIAI	2	2	1	1	3

β2-MG, β2-microglobulin; EB, Epstein–Barr virus; ECOG, Eastern Cooperative Oncology Group Performance status; F, female; IPI, International Prognostic Index; LDH, Lactate dehydrogenase; M, Male; PIAI, Prognostic index for AITL.

### 2.1 Case 1

**Figure 1:**
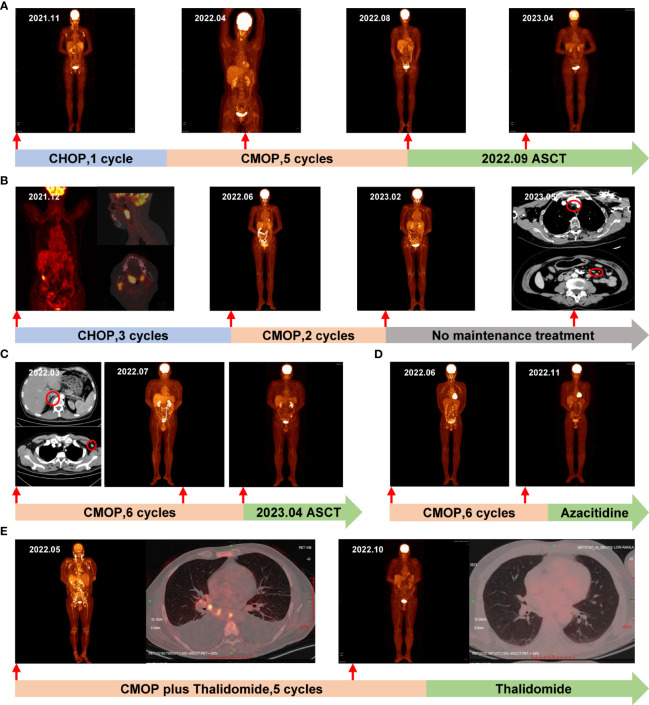
The timeline and representative PET/CT and CT images. Each treatment the patient received is labeled on the graph. **(A)** Case 1: Before treatment in November 2021, PET/CT revealed increased glucose metabolism in multiple areas, including the neck, chest, abdomen, bilateral inguinal lymph nodes, occiput, right chest, back, and subcutaneous fat nodules on both upper extremities. Following 3 cycles of CMOP, a PET/CT scan in April 2022 indicated CR, which was sustained after 5 cycles of CMOP in August 2022. Notably, in April 2023, PET-CT confirmed a lasting CR post-ASCT; **(B)** Case 2: In December 2021, the initial PET/CT revealed enlarged tonsils and right cervical lymph nodes with heightened metabolism. Following 3 cycles of CHOP, a PET/CT scan in June 2022 confirmed CR. Subsequently, after 2 cycles of CMOP in February 2023, another PET/CT displayed CR. However, a CT scan in May 2023 showed enlarged lymph nodes in the mediastinum, and abdominal cavity, marked by red circles; **(C)** Case 3: In March 2022, pre-treatment CT scans revealed enlarged lymph nodes and a nodule on the right side of the thoracic 12 vertebrae, marked by red circles. Subsequently, a PET/CT scan in July 2022 confirmed CR after 4 cycles of CMOP. Another PET/CT in November 2022 validated sustained CR following 6 cycles of CMOP; **(D)** Case 4: In June 2022, pre-treatment PET/CT revealed multiple lymph nodes in the neck, chest, and abdomen. Subsequent PET/CT in November 2022 demonstrated CR after 4 cycles of CMOP; **(E)** Case 5: In May 2022, PET/CT revealed increased glucose metabolism in multiple lymph nodes, bones, and an enlarged spleen, along with pleural effusion. By October 2022, after 4 cycles of CMOP plus thalidomide, PET/CT confirmed CR and the pleural effusion had resolved. PET, positron emission tomography; CT, computed tomography; CR, complete remission; ASCT, autologous stem cell transplantation.

A 54-year-old female diagnosed with stage IVB AITL underwent an 18F-fluoro-2-deoxy-D-glucose positron emission tomography (18F-FDG PET/CT) scan, revealing maximum standardized uptake values (SUV_max_) values of 7.05 in multiple lymph nodes and subcutaneous lesions (**Figure 1A**). The patient had a medical history of hepatitis B. After one cycle of CHOP induction therapy, Case 1 exhibited a suboptimal response with no significant changes in lymph nodes and was later switched to the CMOP regimen (cyclophosphamide, 750 mg/m^2^, day 1; PLM60, 20 mg/m^2^, day 1; vincristine, 1.4 mg/m^2^, day 1; prednisolone, 100 mg/day, days 1-5) on February 2022. The outcome was favorable following one cycle of CMOP therapy, no palpable lymphadenopathy was observed. Three cycles later, a positron emission tomography/computed tomography (PET/CT) scan showed no evidence of residual active tumor tissue, indicating a CR (**Figure 1A**). The patient underwent a total of 5 cycles of CMOP therapy until July 2022. Afterward, autologous stem cell transplantation (ASCT) was successfully performed on September 30, 2022. This patient remained alive with a CR during the 19-month follow-up (**Figure 1A**).

### 2.2 Case 2

A 66-year-old female presented with tonsillar swelling and was diagnosed with stage II AITL. PET/CT showed enlargement of bilateral tonsils and right cervical lymph nodes with increased metabolic activity (SUV_max_, 13.8; **Figure 1B**). Following 1 cycle of CHOP, the patient showed no evidence of this enlargement but developed sick sinus syndrome, and paroxysmal atrial fibrillation (AF). Consequently, the patient underwent permanent dual-chamber pacemaker implantation and then completed an additional 2 cycles of CHOP chemotherapy and achieved a CR (**Figure 1B**). However, due to the ongoing cardiac complications, the treatment approach shifted from the CHOP regimen to the CMOP regimen (cyclophosphamide, 640 mg/m^2^, day 1; PLM60, 18 mg/m^2^, day 1; vindesine, 3 mg/m^2^, day 1; prednisolone, 100 mg/day, days 1-5). Case 2 received 2 cycles of CMOP from June to July 2022, resulting in effective management of atrial fibrillation and achieving CR (**Figure 1B**). She did not enter maintenance treatment because of financial problems. In May 2023, CT revealed enlarged cervical, mediastinal, bilateral hilar, and abdominal lymph nodes. The first-line treatment achieved a PFS of 11 months.

### 2.3 Case 3

A 53-year-old male presented with a left neck mass in 2022. Enhanced CT revealed significantly enlarged left axillary lymph nodes, multiple neck masses, pharyngeal soft tissue thickening, and nodules on the right side of the 12th thoracic vertebrae (**Figure 1C**). Lymph node biopsy confirmed stage IIA AITL. Considering the presence of the mass, 6 cycles of CMOP chemotherapy (cyclophosphamide, 750 mg/m^2^, day 1; PLM60, 16 mg/m^2^, day 1; vindesine, 3 mg/m^2^, day 1; prednisolone, 100 mg/day, days 1-5) was administered as the initial therapy from March to September 2022. In July 2022, the patients who received 4 cycles of CMOP exhibited CR (**Figure 1C**). Subsequently, on April 14, 2023, Case 3 successfully underwent ASCT. During the 16-month follow-up period, the patients demonstrated no evidence of tumor recurrence.

### 2.4 Case 4

A 54-year-old male with generalized papules, pruritus, and multiple enlarged lymph nodes with nocturnal night sweats was diagnosed with stage IIIB AITL. PET/CT indicated that lymphoma involved multiple lymph nodes in the neck, chest, and abdomen with the largest lymph node measuring 22×15 mm and the SUV_max_ of 12.87 (**Figure 1D**). From July to December 2022, he received 6 cycles of CMOP chemotherapy (cyclophosphamide, 750 mg/m^2^, day 1; PLM60, 18 mg/m^2^, day 1; vincristine, 1.4 mg/m^2^, day 1; prednisolone, 100 mg/day, days 1-5) as an initial therapy due to the described symptoms. PET/CT in November 2023 showed CR following 4 cycles of CMOP (**Figure 1D**). Subsequently, he received azacitidine maintenance therapy following the CMOP regimen and has remained in remission, with a follow-up time of 12 months.

### 2.5 Case 5

**Figure 2:**
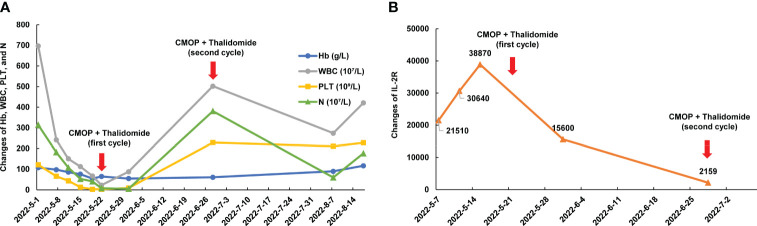
Dynamic changes of blood cell count, ferritin, and IL-2R of case 5 during treatment. **(A)** Dynamic changes of Hb, WBC, PLT, and N during treatment; **(B)** Dynamic changes of IL-2R. Hb, hemoglobin; WBC, white blood cells; PLT, platelets; N, neutrophils; IL-2R, interleukin 2 receptor.

A 52-year-old man, with a history of AF with underlying cardiac disease, was admitted to our hospital for rash and generalized pain and was diagnosed as AITL. Physical examination revealed the diffuse maculopapular rash, markedly enlarged superficial lymph nodes, abdominal distension, and pitting edema in the lower limbs. A hypermetabolic signal was observed from multiple lymph nodes, bones, and an enlarged spleen on PET/CT (**Figure 1E**). Bone marrow examination revealed T lymphocyte infiltrates. Additionally, Case 5 exhibited symptoms including pancytopenia, low levels of fibrinogen, and high levels of soluble interleukin 2 receptor (IL-2R), suggesting a potential diagnosis of hemophagocytic syndrome (**Figure 2**). On May 15, 2022, the therapy including dexamethasone (15 mg/day), etoposide (50 mg/m^2^ on day 2), and thalidomide (100 mg/day) was initiated based on the clinical data. Despite the initial therapy, the condition of Case 5 worsened with severe pancytopenia, edema, pleural and abdominal effusion, and dyspnea, possibly due to lymphoma. Considering less cardiotoxic side effects and more efficacy than anthracyclines, 1 cycle of low-dose CMOP-like regimen (dexamethasone, 15 mg, days 1-9, and 10 mg, days 10-14; vincristine, 0.68 mg/m^2^, days 2-3; cyclophosphamide, 440 mg/m^2^, day 4; PLM60, 10 mg/m^2^, day 7) plus thalidomide (100 mg once daily) along with symptomatic support was administered. After 1 cycle of treatment, the symptoms and signs of the patient decreased significantly, such as dyspnea, edema, and rash, indicating that this treatment plan can effectively relieve symptoms. His general condition markedly improved, with normalized blood counts and decreased IL-2R levels (**Figure 2**). Therefore, Case 5 received additional 4 cycles of CMOP (cyclophosphamide, 525 mg/m^2^, day 1; PLM60, 17 mg/m^2^, day 1; vincristine, 1.4 mg/m^2^, day 1; prednisolone, 100 mg, days 1-5) plus thalidomide (100 mg once daily). Following the fourth cycle of CMOP plus a thalidomide regimen, Case 5 achieved a CR at PET/CT (**Figure 1E**). This patient pertaining to financial constraints was not able to go for an ASCT and hence opted for maintenance therapy with thalidomide. On a 14-month follow-up, his disease continued to be in complete remission.

All five cases exhibited good tolerance to CMOP, experiencing manageable hematological toxicity. Grade 1 skin hyperpigmentation, manifested as bluish staining of the skin, was observed in all five cases, which recovered spontaneously at the end of chemotherapy. No adverse reactions such as infection, stomatitis, or neurotoxicity were observed. In particular, all patients had no new cardiac adverse events during chemotherapy with the CMOP regimen, even though two patients had a previous history of cardiac-related disease (**Table 2**).

**Table 2:** Toxicities in the five cases with CMOP.

Adverse event	Grade 1 or 2	Grade 3 or 4	Total
Leukopenia	3 (60%)	1 (20%)	4 (80%)
Neutropenia	3 (60%)	1 (20%)	4 (80%)
Thrombocytopenia	1 (20%)	0	1 (20%)
Anemia	2 (40%)	1 (20%)	3 (60%)
Nausea	5 (100%)	0	5 (100%)
Vomiting	1 (20%)	0	1 (20%)
Hepatotoxicity	1 (20%)	0	1 (20%)
Hyperpigmentation	5 (100%)	0	5 (100%)

All data was shown as the number of patients.

## 3 Discussion

AITL, a unique subtype of lymphoma, presented challenges in treatment due to its complex nature and comparatively poorer prognosis (12). Conventional anthracycline-based chemotherapy remained a frequently utilized initial treatment option with suboptimal CR rates of 36.6%-47.9% (13) and 5-year OS rates of 43%-50% (2). To enhance the efficacy of conventional treatments, various strategies have been explored, including the addition of other potentially effective drugs, such as adding lenalidomide (14), everolimus (15), or romidepsin (16) to CHOP and brentuximab vedotin plus CHP (cyclophosphamide, doxorubicin, and prednisone) (17). However, these above regimens are still in clinical trials with the absence of recommended standard of care for AITL.

PLM60 has demonstrated promising efficacy and tolerance in PTCL in a previous phase II study (11), providing a new direction in treating PTCL. Here, we reported that 5 AITL patients achieved CR following the CMOP regimen containing the PLM60. To the best of our knowledge, this is the first report on the successful utilization of the CMOP regimen in AITL in the real world. Case 1, who exhibited poor response to CHOP, achieved CR upon switching the CMOP regimen and attained long-term remission following transplantation. In addition, Case 5 had a critical condition with a large tumor burden and hemophagocytic syndrome during the initial treatment; however, rapid improvement was observed after the COMP regimen. These 2 successful cases suggested that the CMOP regimen containing PLM60 may rapidly reduce the tumor burden and promote the remission of the disease, which needed further clinical research and verification.

Post-CR treatment was considered crucial in enhancing long-term survival benefits. In contrast with the non-ASCT group, the ASCT group improved the 2-year OS rates (93.3% vs. 52.9%) for patients with AITL in the first complete remission in a previous study (18). Additionally, results of a meta-analysis showed that ASCT significantly improved OS for AITL patients, indicating ASCT could serve as the first-line consolidation treatment strategy for AITL patients (19). A similar pattern was observed in our cases, with 4 patients who underwent ASCT or pharmacologic maintenance therapy and remained in sustained remission. The lone patient who experienced recurrence did not receive these therapies. Therefore, we recommend that AITL patients, who achieved a CR after chemotherapy, undergo the ASCT as first-line consolidation therapy for long-term clinical benefits.

Mitoxantrone, as a conventional anthracycline, has been demonstrated to be associated with an increased risk of cardiac dysfunction and secondary leukemia (10). In a previous study, compared to the mitoxantrone group, the incidence of increased cardiac troponin T (3.3% vs. 36.7%) was lower in the PLM60 group (20), indicating that the risk of cardiotoxicity with PLM60 was much lower than that with mitoxantrone. The safety result of our cases was consistent with the previous study with mild cardiotoxicity, no serious adverse events, and no dose reduction due to adverse events. Notably, 2 patients with underlying cardiac diseases tolerated PLM60 well. The mechanism of the reduced cardiotoxicity of PLM60 might be attributed to its lower peak concentrations, prolonged half-life, and reduced heart tissue distribution (8, 21). Overall, PLM60 may provide a more tolerable treatment option by potentially redefining the balance between efficacy and safety in AITL treatment.

There are some limitations to our study. First, our study is limited by the small sample size. Second, as the first study of CMOP, we lack comparative data to position our findings against other treatments definitively.

## 4 Conclusions

In conclusion, the CMOP regimen containing PLM60 may provide a novel option for AITL with promising efficacy and safety. While these results of our cases are highly encouraging, further clinical studies are imperative to validate its benefits comprehensively. Several ongoing clinical trials are investigating the effect of PLM60 plus other agents for newly diagnosed PTCL (NCT05458180) and relapsed and refractory PTCL (NCT05441761, ChiCTR2200065840, NCT05527275, ChiCTR2200062067, etc.), showing the potential in the development of combination therapy.

## Data availability statement

The original contributions presented in the study are included in the article/supplementary material. Further inquiries can be directed to the corresponding author.

## Ethics statement

The studies involving humans were approved by Ethics Committee on Biomedical Research, West China Hospital of Sichuan University. The studies were conducted in accordance with the local legislation and institutional requirements. Written informed consent for participation in this study was provided by the participants’ legal guardians/next of kin. Written informed consent was obtained from the individual(s) for the publication of any potentially identifiable images or data included in this article.

## Author contributions

LL: Writing – original draft, Writing – review & editing, Data curation. MJ: Writing – review & editing.
